# Large‐scale collaboration in ENIGMA‐EEG: A perspective on the meta‐analytic approach to link neurological and psychiatric liability genes to electrophysiological brain activity

**DOI:** 10.1002/brb3.2188

**Published:** 2021-07-21

**Authors:** Dirk J. A. Smit, Ole A. Andreassen, Dorret I. Boomsma, Scott J. Burwell, David B. Chorlian, Eco J. C. de Geus, Torbjørn Elvsåshagen, Reyna L. Gordon, Jeremy Harper, Ulrich Hegerl, Tilman Hensch, William G. Iacono, Philippe Jawinski, Erik G. Jönsson, Jurjen J. Luykx, Cyrille L. Magne, Stephen M. Malone, Sarah E. Medland, Jacquelyn L. Meyers, Torgeir Moberget, Bernice Porjesz, Christian Sander, Sanjay M. Sisodiya, Paul M. Thompson, Catharina E. M. van Beijsterveldt, Edwin van Dellen, Marc Via, Margaret J. Wright

**Affiliations:** ^1^ Department of Psychiatry Amsterdam Neuroscience Amsterdam UMC University of Amsterdam Amsterdam The Netherlands; ^2^ Norwegian Centre for Mental Disorders Research (NORMENT) Oslo University Hospital Oslo Norway; ^3^ Institute of Clinical Medicine University of Oslo Oslo Norway; ^4^ Department of Biological Psychology Vrije Universiteit Amsterdam Amsterdam The Netherlands; ^5^ Department of Psychology Minnesota Center for Twin and Family Research University of Minnesota Minneapolis MN USA; ^6^ Department of Psychiatry University of Minnesota Minneapolis MN USA; ^7^ Henri Begleiter Neurodynamics Laboratory Department of Psychiatry Downstate Health Sciences University Brooklyn NY USA; ^8^ Department of Neurology Oslo University Hospital Oslo Norway; ^9^ Department of Otolaryngology Vanderbilt University Medical Center Nashville TN USA; ^10^ Vanderbilt Genetics Institute Vanderbilt University Medical Center Nashville TN USA; ^11^ Vanderbilt Brain Institute Vanderbilt University Nashville TN USA; ^12^ Department of Psychiatry, Psychosomatics, and Psychotherapy Goethe Universität Frankfurt am Main Frankfurt Germany; ^13^ Department of Psychiatry and Psychotherapy University of Leipzig Medical Center Leipzig Germany; ^14^ LIFE ‐ Leipzig Research Center for Civilization Diseases Universität Leipzig Leipzig Germany; ^15^ IU International University Erfurt Germany; ^16^ Department of Psychology University of Minnesota Minneapolis MN USA; ^17^ Department of Psychology Humboldt‐Universität zu Berlin Berlin Germany; ^18^ TOP‐Norment Institute of Clinical Medicine University of Oslo Oslo Norway; ^19^ Department of Clinical Neuroscience Centre for Psychiatric Research, Karolinska Institutet & Stockholm Health Care Services Stockholm Region Stockholm Sweden; ^20^ Department of Psychiatry UMC Utrecht Brain Center University Medical Center Utrecht Utrecht University Utrecht The Netherlands; ^21^ Department of Translational Neuroscience UMC Utrecht Brain Center University Medical Center Utrecht Utrecht University Utrecht The Netherlands; ^22^ Outpatient Second Opinion Clinic GGNet Mental Health Apeldoorn The Netherlands; ^23^ Psychology Department Middle Tennessee State University Murfreesboro TN USA; ^24^ Literacy Studies Ph.D. Program Middle Tennessee State University Mufreesboro TN USA; ^25^ QIMR Berghofer Medical Research Institute Herston QLD Australia; ^26^ Department of Psychiatry State University of New York Downstate Health Sciences University Brooklyn NY USA; ^27^ Department of Psychology Faculty of Social Sciences University of Oslo Oslo Norway; ^28^ Department of Clinical and Experimental Epilepsy UCL Queen Square Institute of Neurology London UK; ^29^ Chalfont Centre for Epilepsy Chalfont‐St‐Peter UK; ^30^ Imaging Genetics Center, Mark and Mary Stevens Neuroimaging and Informatics Institute Keck School of Medicine University of Southern California Marina del Rey CA USA; ^31^ Department of Psychiatry Department of Intensive Care Medicine, Brain Center University Medical Center Utrecht Utrecht University Utrecht The Netherlands; ^32^ Brainlab‐Cognitive Neuroscience Research Group Department of Clinical Psychology and Psychobiology, and Institute of Neurosciences (UBNeuro) Universitat de Barcelona Barcelona Spain; ^33^ Institut de Recerca Sant Joan de Déu (IRSJD) Esplugues de Llobregat Spain; ^34^ Queensland Brain Institute University of Queensland Brisbane QLD Australia; ^35^ Centre for Advanced Imaging University of Queensland Brisbane QLD Australia

**Keywords:** brain disorders, electroencephalography, ENIGMA, harmonization, imaging genetics, open science

## Abstract

**Background and purpose:**

The ENIGMA‐EEG working group was established to enable large‐scale international collaborations among cohorts that investigate the genetics of brain function measured with electroencephalography (EEG). In this perspective, we will discuss why analyzing the genetics of functional brain activity may be crucial for understanding how neurological and psychiatric liability genes affect the brain.

**Methods:**

We summarize how we have performed our currently largest genome‐wide association study of oscillatory brain activity in EEG recordings by meta‐analyzing the results across five participating cohorts, resulting in the first genome‐wide significant hits for oscillatory brain function located in/near genes that were previously associated with psychiatric disorders. We describe how we have tackled methodological issues surrounding genetic meta‐analysis of EEG features. We discuss the importance of harmonizing EEG signal processing, cleaning, and feature extraction. Finally, we explain our selection of EEG features currently being investigated, including the temporal dynamics of oscillations and the connectivity network based on synchronization of oscillations.

**Results:**

We present data that show how to perform systematic quality control and evaluate how choices in reference electrode and montage affect individual differences in EEG parameters.

**Conclusion:**

The long list of potential challenges to our large‐scale meta‐analytic approach requires extensive effort and organization between participating cohorts; however, our perspective shows that these challenges are surmountable. Our perspective argues that elucidating the genetic of EEG oscillatory activity is a worthwhile effort in order to elucidate the pathway from gene to disease liability.

## Graphical abstract

We present the perspective of the ENIGMA‐EEG workgroup on our goal to elucidate the genetics of brain activity measured by electroencephalography. We explain the issues that arise when performing an international multicohort study and the solutions to these problems. We summarize our initial results and lay out our plans for future electroencephalographic features that are crucial to investigate in order to link liability genes to brain activity.

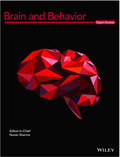

## 1 INTRODUCTION

The ENIGMA‐EEG working group was established to enable large‐scale international collaborations among cohorts who investigate the genetics of brain function measured with electroencephalography (EEG). EEG has been used for many decades to investigate cognitive processes and individual differences in brain function and to discover biomarkers for neurological, psychiatric, sleep, and other disorders (Berry et al., 2017; Hughes & John, 1999; Noachtar & Rémi, 2009). Until the advent and widespread use of functional MRI, EEG was the primary method for measuring activity of the brain, but has remained an important part of neuroscientific research. EEG can directly measure synaptic processes with higher temporal resolution (in the millisecond range), which makes it different from other imaging modalities like functional MRI. It is also silent and more comfortable for the participant—that is, less intrusive, and less cramped and noisy—thus affecting the subject less than functional MRI during the recordings. It is also much less expensive and more convenient to use, making it feasible for widespread research and clinical application worldwide.

EEG research has a rich history of providing biomarkers for behavioral traits and mental health disorders. The primary interest of ENIGMA‐EEG is, however, not to repeat biomarker research in larger samples. Individual variation in many of the EEG biomarkers has been found to be under substantial genetic control, with twin and family studies provided the crucial information that EEG trait variation is heritable. Early studies, dating back to the 1930s, pointed toward nearly identical recordings of resting EEG in identical twins (reviewed in van Beijsterveldt and Boomsma (1994)). The first large‐scale twin studies carried out by Friedrich Vogel (1958); described in Vogel (1970) demonstrated that differences between monozygotic twins did not exceed those seen in successive EEG recordings from the same individual, leading to the conclusion that variability in EEG features is nearly completely determined by a multifactorial genetic system. Subsequent studies of other resting EEG features in children, adolescents, and adults showed that EEG measures of oscillation power, oscillation dynamics, and connectivity are heritable traits (Anokhin et al., 2001; van Beijsterveldt & van Baal, 2002; van Beijsterveldt & van Baal, 2002; Chorlian et al., 2007; Posthuma et al., 2005; Rangaswamy & Porjesz, 2008; Smit et al., ,,^2005^, ^2010^; Tang et al., 2007; Zietsch et al., 2007).

At the same time, it is well known that the liability for neurological and psychiatric disorders is under genetic control (Polderman et al., 2015). In the beginning of this century, many argued that investigating EEG as an intermediate phenotype (or endophenotype) would aid in finding specific genes for behavioral traits and mental health disorders (de Geus, 2010). This idea was surpassed by the massive case–control genome‐wide association studies (GWAS) performed in human genetics (Bulik‐Sullivan et al., 2015; Sullivan 2010). However, the black box method of GWAS leaves unexplained how specific risk variants exert their influence on the brain on a systems level (de Geus, 2010). This is the main focus of our consortium: to find how genetic variants influence individual variation in EEG phenotypes and to link these to variants affecting brain disorders.

**FIGURE 1:**
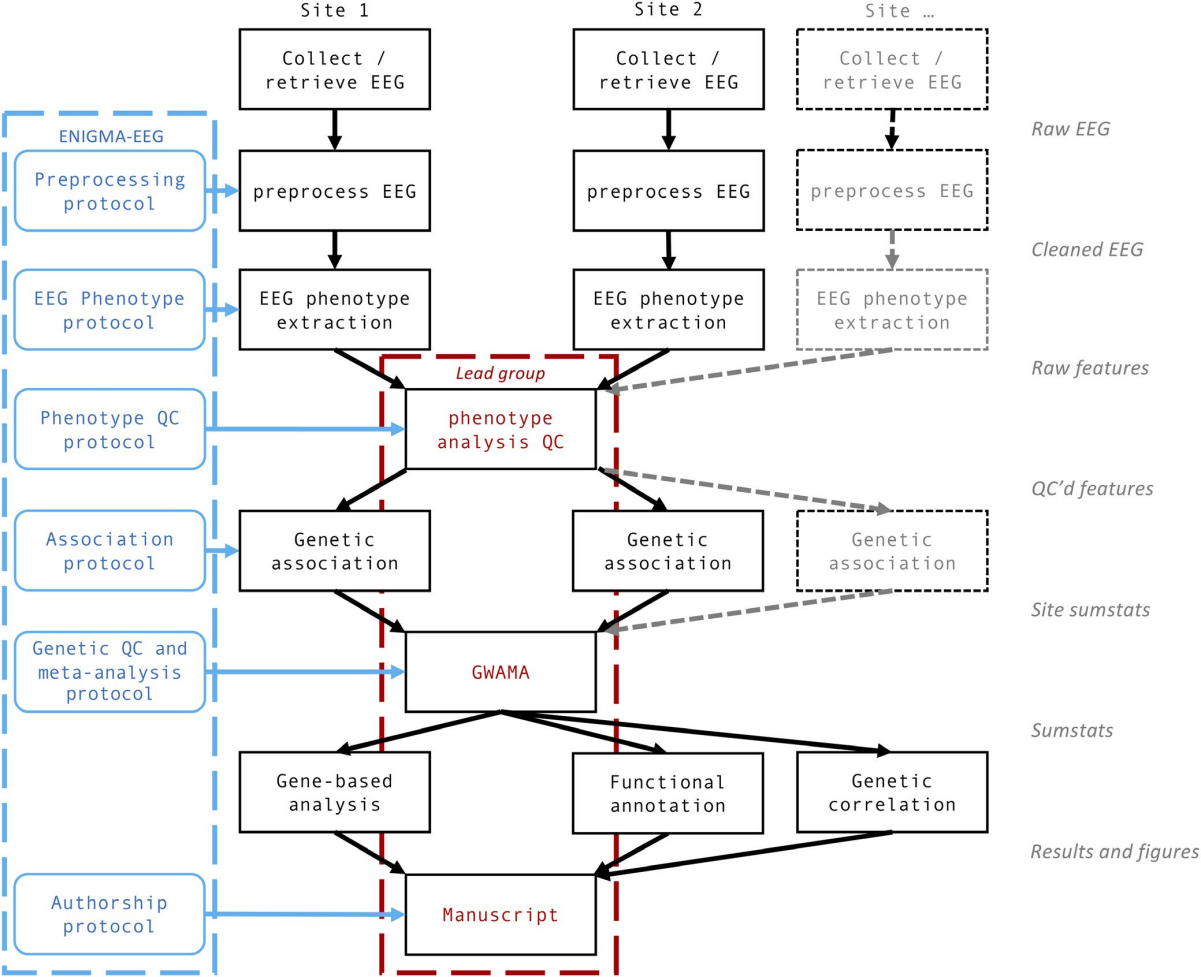
The organization of the work required in our investigations of EEG genetics. Much of the work is performed by the collaborating sites (columns in black), including EEG recording, preprocessing, phenotype extraction, and performing the genetic association. The role of ENIGMA‐EEG is to regularly hold teleconference calls to create the protocols for EEG analysis, QC, and genetics analyses (blue). Lead groups of ENIGMA‐EEG members are formed to perform centralized quality control (QC) of the EEG features and to meta‐analyze of the summary statistics provided by the sites. The summary statistics are then distributed to the individuals who will perform genetic follow‐up analyses. Finally, a manuscript is prepared. Note that most of the genetics work is not included in this workflow, thus excluding a huge amount of work on taking biological samples (blood, saliva), DNA extraction and storage, sending for genotyping, data management, imputation, quality control. EEG, electroencephalography; ENIGMA, Enhancing NeuroImaging Genetics through Meta‐Analysis; GWAMA, genome‐wide meta‐analysis; QC, Quality control; Sumstats, Genetic summary statistics from genome‐wide association

In order to do so, we need to increase sample sizes sufficiently to reach the statistical power required to detect and replicate genetic associations of common variants with the EEG biomarkers. Genetic variants typically have small effect sizes, thus requiring large sample sizes. As in other ENIGMA workgroups, a core element of ENIGMA‐EEG is to perform our genetic studies using a meta‐analytic approach, where participating cohorts analyze their data locally, after which they are collected, scrutinized, and meta‐analyzed, after which they are linked to the disorders. This requires coordination between participating cohorts in prioritizing subject of investigation, the method of analysis, and coordination of the effort put in by each site to reach the inevitable goal of science, the manuscript. Figure 1 shows the workflow of our consortium in more detail, illustrating that collaborative efforts require extensive discussion and coordination. It shows how data/results are shared, what data/results are shared, the role of each of the participating sites, and the role of ENIGMA‐EEG in coordinating this process. As Figure 1 shows, most of the work is performed by the collaborating sites, including EEG recording, preprocessing, phenotype extraction, and performing the genetic association. The role of ENIGMA‐EEG is to regularly hold teleconference calls to discuss progress, provide support, and make decisions that lead to the analysis plan. In doing so, we are supported by ENIGMA (Thompson et al., 2014, 2017), who provide the infrastructure for teleconferencing, sharing protocols, and results (http://enigma.ini.usc.edu/). ENIGMA holds annual meetings (virtual or live) to provide a platform for collaborations between the workgroups, for sharing methods or any other type of collaboration (e.g., between ENIGMA‐EEG and ENIGMA‐MEG, ENIGMA Epilepsy, and ENIGMA Genetics).

In the following sections, we show our perspective on how to tackle the open questions in the field of EEG genetics. We first argue why EEG may be crucial for advancing understanding of synaptic and circuit‐level functioning of the brain in normal functioning and disease, and how oscillatory activity captures important characteristics of information processing in the brain. Next, we focus on describing the challenges for international EEG genetics collaborations, especially those regarding analytic and methodological choices, and homogeneity within and across cohorts. We also describe in more detail the key scientific questions that we aim to address in our next endeavors. This results in our future plans for investigating the genetics of EEG‐based brain activity.

## 2 A FOCUS ON EEG OSCILLATIONS IN NEURAL PROCESSING

EEG has provided the scientific community with a large range of biomarkers and putative biomarkers for neurological and behavioral disorders, offering insight into the localization and timing of cognitive processes (Arns et al., 2013; de Geus, 2010; Hegerl et al., 2008; Murias et al., 2007; Uhlhaas & Singer, 2010), and has provided biomarkers that track brain development (Smit & Anokhin, 2016; Smit et al., 2011, 2012). More recently, EEG has provided insight into modes of communication in large‐scale brain networks via synchronous oscillatory activity (Cohen et al., 2012; Horschig et al., 2015; Salinas & Sejnowski, 2001; Stam, 2014; Uhlhaas et al., 2010; Varela et al., 2001). For clinical purposes, EEG is routinely used in the diagnosis of neurological disorders. It is the gold standard for sleep staging, which is used to establish disruption of sleep patterns (Berry et al., 2017; Coleman et al., 1982). EEG is also used to detect epileptiform activity and epileptic seizures or their absence (Flink et al., 2002; Niedermeyer, 1999a), or to monitor nonconvulsive status epilepticus in critically ill patients (Abend et al., 2010). By contrast, EEG has only rarely found a way into clinical use for diagnosis and evaluation of psychiatric disorders, although complementary treatments that use EEG are being widely offered in the form of neurofeedback training (Enriquez‐Geppert et al., 2017). Many patents have been filed for diagnostics or neurofeedback systems (patent category A61B5/0482), but only a single method has achieved FDA approval (theta/beta ratio for ADHD; see Arns et al., 2016).

EEG recordings from the scalp as well as intracranial recordings indicate that cortical communication is the result of neuronal oscillations (Akam & Kullmann, 2014; Cohen, 2017; Stam, 2014; Uhlhaas et al., 2010; Uhlhaas & Singer, 2010). Both intracranial and scalp recordings show clear sinusoidal activity patterns, produced by the oscillations that are caused by the concerted changes in voltages across the postsynaptic membranes in the dendritic trees of pyramidal neurons. These voltage changes either inhibit or sensitize neurons to create action potentials. The unique orientation of the pyramidal neurons—systematically perpendicular to the cortical sheet—and the often strongly correlated coactivations across many neurons result in a large collective dipole: the summed activity of the local field potentials that is enough to propagate through the surrounding tissues to reach externally attached electrodes (Buzsáki et al., 2012). Although the activity on the microscopic scale of a single neuron cannot be detected, there is enough information for recording synchronous activity of patches of neurons, at the scale of a few cm^2^ of cortical tissue.

Our primary focus is to analyze this oscillatory activity—mostly from the eyes‐closed resting state, the commonest condition applied during EEG acquisition. From these recordings, it is possible to extract a near endless set of EEG features based on oscillatory frequency, oscillatory power, the temporal dynamics of oscillations, interactions across oscillatory frequencies, and spatial interactions (connectivity). In the upcoming sections, we will highlight the strategic selection of EEG variables. Oscillatory responses to events and event‐related potentials (ERPs) in task data are another great source of clinical biomarkers (Duncan et al., 2009). Most notably, ERP components such as the P300 obtained in the oddball task, the mismatch negativity, and the steady‐state responses are among the most relevant for clinical research. While ERPs are not reviewed in the current perspective, they will be targeted in future work by our consortium.

Oscillations are not epiphenomenological features of brain activity, but have functional relevance. Their synchronicity is a main mode of meso‐ and long‐distance communication between cortical areas and from cortical to subcortical areas (Fries, 2005; Horschig et al., 2015; Schnitzler & Gross, 2005). Coactivation in the form of spike propagation can only take place when brain areas are in synchrony, that is, are both sensitive to input in a depolarizing phase. Different oscillation frequencies subserve different cognitive and affective functions, while sharing the same anatomical network (Akam & Kullmann, 2014; Klimesch, 1996). Such communication has proven essential for executing a wide range of behavioral tasks, may also be affected in behavioral disorders, and may to some extent explain individual differences in behavior (Arns et al., 2013; Doppelmayr et al., 2002; Jenkinson & Brown, 2011). The causal role neural oscillations play in behavioral variation is becoming increasingly clear. For example, blocking beta oscillations (13–30 Hz) in the subthalamic nucleus (STN) of patients with Parkinson's disease during deep brain stimulation treatment can result in relieving bradykinetic symptoms (Engel & Fries, 2010; Swann et al., 2011); moreover, stimulation only during periods of elevated beta activity in the STN is sufficient to obtain symptom relief (Little & Brown, 2014). This finding led to the conclusion that a surplus STN beta activity causes bradykinesia (Brown, 2006). Other examples using optogenetic driving of oscillations in the mouse brain further highlight the contribution of oscillatory activity to communication and behavior (Cho et al., 2015; Karalis et al., 2016).

A further reason to investigate neural oscillations is that they have been investigated well in the extant literature. Many of the participating cohorts were established long ago as (twin) family study cohorts.

## 3 INITIAL ENIGMA‐EEG FINDINGS

ENIGMA‐EEG published their first article in November 2018 on the genetics underlying the strength of oscillations present in EEG brain activity signals (Smit et al., 2018). We associated genome‐wide SNPs to oscillation strength in the common delta, theta, alpha, and beta frequency bands, and alpha peak frequency. All these brain activity traits are under moderate to strong genetic control and are to some degree biomarkers of behavioral traits and liability to psychiatric illnesses (Boutros et al., 2008; Klimesch, 1996; Porjesz & Begleiter, 2003). Our primary aim in this project was to increase power to find genetic associations by increasing sample size. The sample size of previous studies was modest, with the largest study analyzing a sample of just over 4,000 individuals (Malone et al., 2014). Malone et al. did not find any significant individual SNPs, but detected associations at the gene level for delta power.

Our study of EEG data from 8,425 individuals found genome‐wide significant hits—that is, genetic variants associated with EEG signal variation—although these did not remain significant when correcting across the various EEG phenotypes tested (i.e., the five oscillation frequency powers and alpha peak frequency). The association results are available upon request via http://enigma.ini.usc.edu/ongoing/enigma‐eeg‐working‐group/. Our application procedure requires filling out a short form with contact information and requires the requestor to agree with the ethical statement regarding the download of genetic association data. The biological function of the SNPs was investigated using several gene‐based and gene expression‐based approaches. These results highlighted several significant effects across the genome. One important region associated with alpha oscillations was found on 3p21.1, which holds many genes associated with risk for schizophrenia and bipolar disorder (Ripke et al., 2014; Stahl et al., 2019). Brain expression analysis found significant effects on *GNL3* and *ITIH4* expression in prefrontal cortices, explaining the observed aberrant brain activity in schizophrenia. Recently, a study found that the genetic variants in the 3p21.1 region affect expression of *NEK4*, *GNL3*, and *PBRM1* in the frontal cortices, which in turn affected dendritic spines, cognitive function, schizophrenia, and bipolar disorder (Yang et al., 2020). This provides evidence that frontal EEG alpha oscillations may indeed be a biomarker for schizophrenia (Merrin & Floyd, 1996; Nikulin et al., 2012), although the effect has not always been consistent across studies of oscillations at this frequency (Boutros et al., 2008).

### 3.1 Follow‐up inquiries into the genetic contributions to EEG

Based on these genome‐wide association results, several analyses were performed to investigate interesting targets, brain expression of genes, and links to psychiatric and neurological phenotypes. Alcohol dependence has been found to be linked to SNPs in gamma‐aminobutyric acid‐receptor subunit alpha 2 gene (*GABRA2)* (Dick et al., 2006; Edenberg et al., 2004; Porjesz et al., 2002; Rangaswamy & Porjesz, 2008; Rangaswamy et al., 2002). *GABRA2* and alcoholism have been linked to individual differences in beta oscillation power (12–28 Hz) (Edenberg et al., 2004; Porjesz et al., 2002; Rangaswamy & Porjesz, 2008; Rangaswamy et al., 2002). We aimed to replicate this last result using our genome‐wide study of beta oscillation power. The association was found to be present in the gene‐based test (Smit et al., 2018). Gene‐expression analysis of the *GABRA2* gene suggested that beta power was most strongly associated with hippocampal expression. This suggests that hippocampal GABRA2 expression affects beta oscillations and may be linked to the pivotal role hippocampal GABA plays in habit‐forming and reward processing in alcohol dependence (Enoch, 2008). Interestingly, this is in line with ongoing work by ENIGMA's Addiction working group; a fine‐scale morphometric analysis alcohol dependence was associated with abnormalities in a range of structures, but showed the strongest effects in the hippocampus (Chye et al., 2020), as well as in the thalamus, putamen, and amygdala.

In a preprint manuscript (Stevelink et al., 2019), we explored the genetic correlation between theta and beta power and the generalized genetic epilepsy (GGE) GWAS of the International League Against Epilepsy (ILAE Consortium, 2014). Beta power in particular may prove to be a biomarker with links to GABA expression in inhibitory interneurons (Hall et al., 2010; Porjesz et al., 2002; Rangaswamy et al., 2002) and consequently may have a role in epilepsy when inhibition and excitation are imbalanced (Magloire et al., 2019). Beta power is generally not considered interictal epileptiform brain activity, which typically includes spike and sharp wave activity (Pillai & Sperling, 2006). Significant positive genetic correlations were found between beta power and liability for GGE, indicating shared genetic architecture. In an independent Epilepsy GWAS (Epi25 consortium), the genetic correlation remained significant. Since the participants studied in ENIGMA‐EEG were all nonepileptic (epilepsy in all its forms is an exclusion criterion for most if not all EEG studies that do not focus on epilepsy), this provides some insights into whether resting‐state recordings can be used to determine neuronal hyperexcitability below the clinical threshold, which may affect psychological function and explain some of the comorbidities and genetic correlations observed between behavioral disorders and epilepsy (Anttila et al., 2018; Bulik‐Sullivan et al., 2015; Gaitatzis et al., 2004; Hesdorffer et al., 2012; Swinkels et al., 2005; Volkmar & Nelson, 1990).

Despite the modest sample sizes—small when compared to the very large GWASs of psychiatric disorders and other complex traits—our initial GWAS of oscillation strength already found significant loci for alpha band oscillations with plausible biological mechanisms. These associations will continue to be followed up as we further increase our sample sizes. Combining SNP results into gene‐based and gene‐expression tests, we observed significant associations with alpha oscillation strength and pointed to brain areas and genomic loci previously linked to psychiatric disorders. EEG oscillatory parameters may be less polygenic than other complex traits—although not to the degree as previously suggested or hoped for (see also de Geus, 2010). Nevertheless, genetic analyses of EEG features are starting to be helpful in explaining how specific psychiatric liability genes affect the functioning brain, plotting the pathway from SNP to expression to neural‐level function, to systems‐level function, and finally to behavior (de Geus, 2010; Iacono, 2018). Investigating these pathways will be greatly aided by increase sample sizes and by establishing the EEG features' polygenicity (Holland et al., 2019), as well as variant‐level joint‐polygenicity analyses that are currently being developed (Frei et al., 2019) to investigate the nature of overlap between traits (in our case, EEG features and psychiatric/neurological genetic overlap).

## 4 PRACTICAL ISSUES IN HARMONIZING EEG ANALYSES FOR GENETIC ANALYSES

To optimize detection of genetic associations, we can, in addition to increasing sample sizes, invest in harmonizing the phenotype and explore options for multivariate analyses as EEG features are inherently multidimensional. In this article, we highlight our efforts to extract harmonized EEG features, the steps we have taken, and the future steps we would like to take. Equally important is the harmonized analyses of the genetic information. For genotyping, imputation, and quality control of genetic data, we closely follow the recommendations and pipelines from our colleagues in the ENIGMA Genetics working group described online (http://enigma.ini.usc.edu/protocols/genetics‐protocols/) (Grasby et al., 2018; Hibar et al., 2015; Stein et al., 2012). These guidelines and protocols, which we consider just as important as high‐quality neurophysiological biomarker extraction, will not be further covered in the current article, since there is a large specialized body of literature describing QC and methodology for genetic association, genetic meta‐analysis, and polygenic score calculation (e.g., Lam et al., 2020; Marees et al., 2018; Privé et al., 2020; Ni et al., 2021).

Genetic studies typically investigate how individual differences in phenotypes are affected by genetic variants—specifically for our consortium, individual differences in EEG parameters. Therefore, the quality of EEG feature extraction needs to be assessed against the background of the variability of the EEG features in the population being measured. This means that apparatus, data quality control, and sampling/cohort characteristics must not greatly affect the individual participants' rank ordering on the EEG features extracted and should largely capture the same variation. Some aspects of recording are not likely to affect the variability of the EEG features, that is, when they cause a fixed bias. For example, the recording filter settings, with their mostly linear effects on oscillation power and when applied constantly across individuals, will not affect the relative score between individual participants on EEG power. Other aspects of recording, on the other hand, may greatly affect the rank ordering of individual data. For example, if a subset of participants were to fall asleep during the resting recordings, this would greatly affect their average power of oscillatory activity (Niedermeyer, 1999b). To avoid such problems, strict protocols are needed to prevent participants from falling asleep. Experience teaches us that—for younger participants—it is wise to record shorter intervals in the eyes‐closed resting state, as they tend to fall asleep faster than adults. Many more challenges exist in the creation of repeatable recordings within individuals and consistent recordings across individuals. This has led to the creation of guidelines for the application, recording, and analysis of EEG data, often with a special focus on clinical recordings (Babiloni et al., 2020; Flink et al., 2002); see https://www.acns.org/practice/guidelines.

### 4.1 Apparatus

The actual recording of scalp potentials—picking up the minute voltages—may be one of the lesser worries for homogeneity across participating cohorts, given today's high‐quality research EEG equipment. Large individual differences in oscillatory amplitudes and other biomarkers exist in EEG signals that will not be affected crucially by amplifier quality, especially when enough data are available per subject for stable estimates. Likewise, active versus passive electrodes—the use of which is generally linked to the choice of apparatus—is not expected to show large effects on EEG parameters as long as dry electrodes are avoided (Laszlo et al., 2014; Mathewson et al., 2017).

There are, however, many other possible sources of heterogeneity that do affect individual scores on EEG features differentially, affecting the rank ordering of individual subject data and, consequently, genetic associations. We identified several sources of heterogeneity that could substantially affect the individual differences in EEG features. These range from methodological systematicity, to analytical systematicity, to sampling variability.

### 4.2 Methodological issues

One particular methodological challenge for our meta‐analytic approach is that not all cohorts use the same electrode layout and reference electrode during recording. From the 1990s onwards, the number of recording channels has steadily increased, with the most recent cohorts measuring at least 30 channels or more. It is hardly debated that increased density provides highly valuable information on individual differences in brain function, in both health and disease. The earlier studies, however, recorded with 5, 7, 14, or 15 channels. Sparse layouts such as these greatly reduce the possibility to provide more localized brain activity measures.

Data cleaning may be a greater challenge, as methods such as independent component analysis (ICA) for removing eye movement and other artifacts from the EEG signal may not be applicable for the sparsest of montages. For the slightly less sparse montages, separating brain from nonbrain source signals in the recorded traces may be difficult. It often involves a comparison of loading patterns of independent components onto nearby electrodes: If source activity is observed on a single EEG electrode, it cannot come from a brain source however close to the skull and dura. Brain sources project their electrical activity to an area of the scalp which generally contains several electrodes in a recording array of 30 channels or more, due to the high relative impedance of the skull compared to the brain and scalp tissues. The denser the electrode layout, the better it can be evaluated whether a source signal stems from a brain source or outside.

Another notable issue is the sampling frequency and the associated change in the low‐pass hardware filter. These filters are implemented with specific hardware (rather than the digital filtering applied in the postacquisition data cleaning phase) and are required to avoid so‐called aliasing effects in the analog‐to‐digital conversion phase where high‐frequency oscillations can be mistaken for low‐frequency oscillations. Unfortunately, these anti‐aliasing hardware filters also affect the oscillatory amplitude and phase of oscillations near the filter boundary, which is set in relation to the sampling frequency. When the sampling rate is too low (e.g., 256 Hz with an anti‐aliasing filter at 64 Hz), this will affect EEG power well below the 64 Hz cutoff frequency. Moreover, causal filters substantially affect the phase of oscillations, which will subsequently affect cross‐frequency amplitude–phase coupling and phase‐locking values. These issues are easily avoided by increasing sampling frequency and the causal low‐pass filter settings.

**FIGURE 2:**
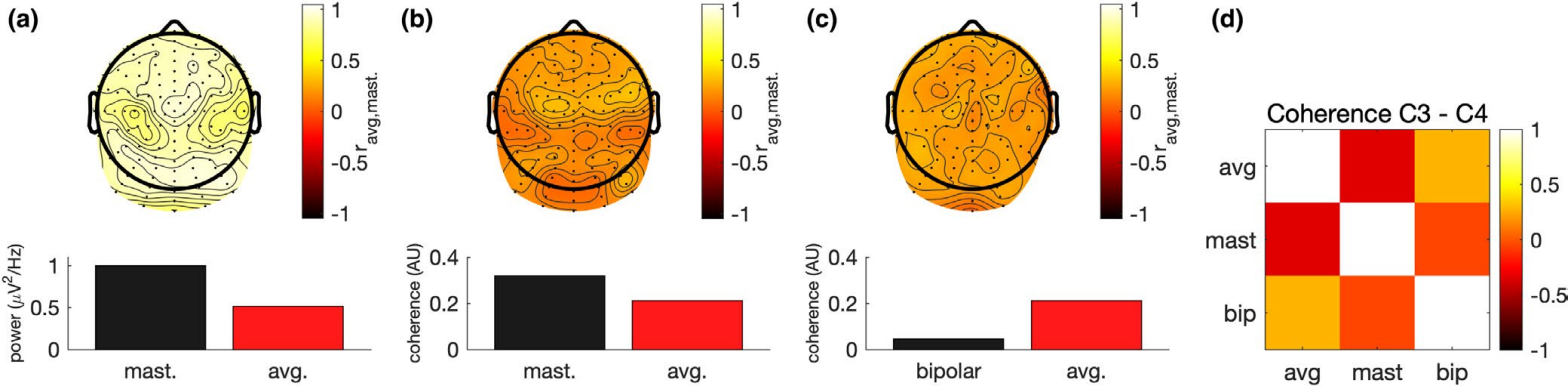
Effect of reference on EEG coherence and power. We calculated power and coherence in the alpha band (8–12.5 Hz) for the 128 channels available in this sample of 39 subjects (data from (Smit et al., 2013)). Data were initially analyzed with average reference. (a) Changing to mastoid reference biases alpha power upward (left inset bar graph). The correlation between mastoid and average reference is very high (>0.90). Therefore, a GWAS of EEG alpha power will be marginally impacted despite the large bias. (b) Changing to mastoid reference also biases channel average coherence upward (inset bar graph). The correlation across subjects is low (*r* < .30, right topoplot). This will substantially affect genetic association and indicates that reference needs to be harmonized across studies. (c) Local bipolar derivations show similar low correlation with the average reference setup (*r* < .28). (d) A selected channel pair (C3, C4) showed variable connectivity between the reference setups. Markedly, mastoid reference showed negative correlation with the average reference and local bipolar derivations

Perhaps the most influential issue with regard to recording is the choice of reference electrode (mastoids, earlobe, nose, average reference, local derivations, dura imaging), affecting how each signal represents shallower and deeper sources. For oscillation power, the effects may be not as crucial, however, for connectivity measures like coherence, substantial effects are expected (Peterson et al., 2019). Figure 2 shows how a change in reference from average to mastoid affects oscillation power and coherence in the alpha band. This required us to make explicit choices in the reference setup to harmonize the results across cohorts for studying the genetics of functional brain connectivity.

Finally, it is widely acknowledged that EEG consists of a myriad of oscillations at various frequencies that all serve different functional purposes (Akam & Kullmann, 2014; Klimesch, 1996). EEG is generally separated into oscillation frequency bands that reflect these functional differences; however, the choice of cutoff frequencies separating these bands is generally taken for granted and reflects commonly accepted fixed definitions. For example, where many studies have captured alpha oscillations as a single entity to be analyzed across the 8–12 Hz frequency band (Palva et al., 2013; Smit et al., 2013), many others have used separate upper and lower bands as they found these to be informative for the functional properties investigated (Doppelmayr et al., 2002; Klimesch et al., 1997; Stam, 2000). Arguably, a frequency band definition can be performed in a more bottom‐up fashion, using the data to optimize information content in the frequency band definition. For our upcoming functional connectivity project, we have used such an approach. The Box 1 below explicates how this analysis was performed, with further specifics provided in the supplementary information. By using this approach, we decrease the heterogeneity induced by suboptimal frequency band definition, while increasing the stability of our estimates and power of our tests by averaging coherence across multiple frequencies.

BOX 1 **Data‐driven frequency band definition for connectivity analysis of EEG**

For our connectivity analysis, we decided to follow a bottom‐up approach to frequency band definitions. For a full description of the methodology, we refer to the online supplementary information. In short, the cutoff frequencies separating the frequency bands were based on the ability of the bands to reproduce the relevant features of the unbanded data (in this specific case, the coherence spectrum at full 0.5 Hz resolution). This was assessed by finding a vector of frequency separation points such that a linear combination of the banded data was best able to reconstruct the unbanded data. The reconstruction fit was measured by the relative matrix distance (Frobenius distance) between the unbanded data and the reconstructed data.

A dataset comprising 240 adult subjects from the COGA cohort (Table 1) was used to calculate coherence between channel pairs at 0.5 Hz resolution (Chorlian et al., 2009). The coherence spectra were limited to 3–28 Hz. An adapted Nelder–Mead function minimization procedure was used to identify optimal separation points between frequency bands using the above criterion of providing the best reconstruction of individual band power, which was then averaged over subjects. This approach yielded separation frequencies as specified in the table. Our empirically derived definition is very near the definitions for theta, lower alpha, upper alpha, lower beta, and upper beta coherence, although specific boundaries deviated slightly from those used regularly. Note that a six band definition was also calculated which provided an additional beta band (see supplement). Only the five‐band definition is shown below.

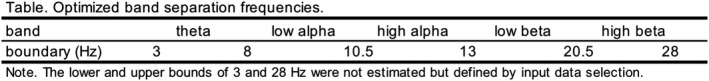

**TABLE 1:** Overview of ENIGMA‐EEG GWAS samples with eyes‐closed resting recordings

Cohort	*N*	Age range (years)	Recorded time (eyes closed)	Number EEG channels	Sampling frequency	Population based/case–control	Dominant ancestry
BATS	971	15.4–19.2	5 min	15	500 Hz	Population based	EUR
COGA	2,835	10.4–74.1	4.25 min	19/31/61	256 Hz	Case–control (alcoholism)	EUR/AFR
LIFE	3,138	41.0–79.9	20 min	30	1,000 Hz	Population based	EUR
MTFS	5,319	16.6–65.3	5 min	5/61	128 Hz	Population based	EUR
NORMENT	416	18–86	5 min	64	2,048 Hz	Case–control (psychotic)	EUR
NTR	839	5.2–70.9	3 min	14/19	250 Hz	Population based	EUR
TSSC	127	5 years–46	2–3 min	128	500 Hz	Population based	EUR
BENEPEG	1,166	≥18 years	3 min	64	500 Hz	Case–control (various psychiatric)	EUR

Abbreviations: BATS, Brisbane Adolescent Twin Study; BENEPEG, Belgium‐Netherlands study of Psychiatric EEG and Genetics cohort; COGA, Collaborative studies on the genetics of alcoholism; LIFE, Leipzig Research Centre for Civilization Diseases; MTFS, Minnesota Twin Family Study; NORMENT, Norwegian Centre for Mental Disorders Research; NTR, Netherlands Twin Register; TSSC, Tennessee Synchrony & Speech Cohort.

### 4.3 Analytic consistency

At ENIGMA‐EEG, our efforts to produce homogenous results for meta‐analysis were mostly focused on analytic techniques and postprocessing of the EEG data. Our customized scripts for extraction of the EEG features were written in MATLAB, passed to participating cohorts, and then applied to the cleaned EEG data. These protocols are available on Github (dirkjasmit/ENIGMA‐EEG).

**FIGURE 3:**
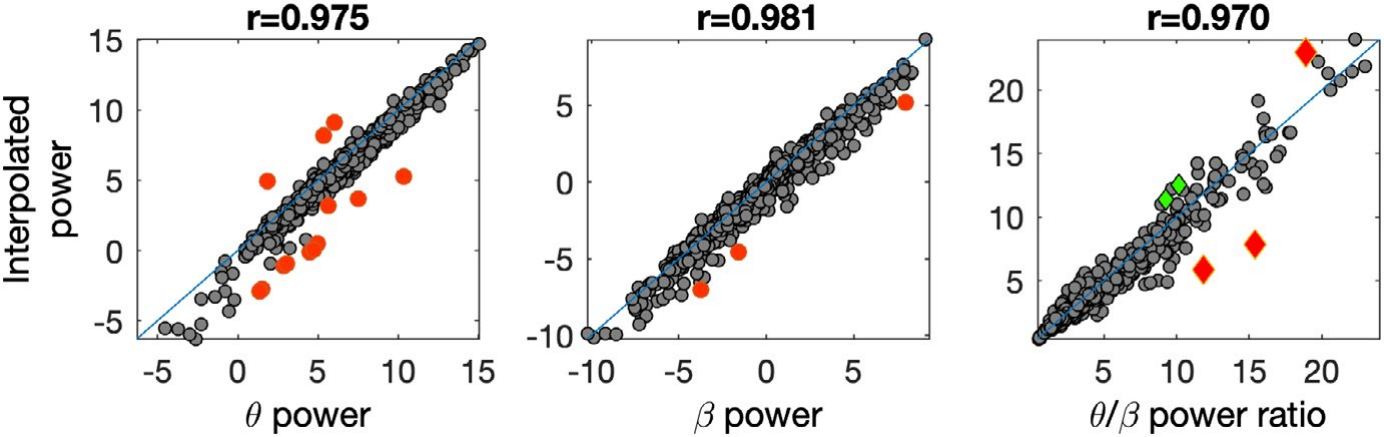
Spherical interpolation for quality control of a dataset of 765 subject in a 17 channel montage with A1/A2 reference using the data from (Smit et al., 2005), eyes‐close resting condition, and cleaned by visual inspection, filtering 1–30 Hz, and ICA decomposition with visual rejection (Pion‐Tonachini et al., 2019). Theta power (4–8 Hz, left), beta power (13–21 Hz, middle), and theta–beta ratio (right) were calculated for channel Cz. Next, the same power values are calculated for a spherical interpolation of channel Cz using 16 remaining channels (implemented in EEGLAB (Delorme & Makeig, 2004)). Even at this low‐density montage, oscillatory power is generally quite well imputed (*r* ≥ .97), and outliers easily detected by statistical methods (false discovery rate). For theta power, ten observations were considered suspect at FDR *q* = 0.01. For beta power, three observations were considered suspect. These values may be replaced with the imputed values. For theta–beta ratio, five values were considered suspect. Retracing the subjects' signals revealed that three of these were affected by some residual artifacts in channel Cz, and their values replaced by the interpolated values. It shows that highly automated algorithms of multichannel EEG data can produce high‐quality data and flag errors in visual cleaning

We are currently extending the EEG feature extraction procedures by providing techniques for postextraction quality control. Although EEG is known for the relative high time investment required to produce clean, artifact‐free stretches of data, it is also quite unique for applying quality checks because of the large number of signals that are recorded from each subject. Each of these signals' extracted parameters can be matched against those of neighboring signals. Using *spherical interpolation*, signals can be recreated based on a fixed weighted average of all remaining electrodes, the EEG feature in question recalculated and matched against the original value (Junghöfer et al., 2000). Alternatively, machine learning can be used to establish an empirically estimated relation between the highly correlated values across the electrode locations and compare the actually obtained values to the values the model imputes from the data. Values with a deviance greater than expected may be removed or replaced by the imputed value. Figure 3 shows an example of how interpolation was used to detect rogue data points in the theta–beta ratio.

Artifact removal from the EEG traces is a constant focus for many EEG researchers. Trained researchers are consistent among each other with an ICC above 0.80 for the extraction of certain power values (Hatz et al., 2015). With the increasingly expanding number of datasets, much effort is being put into automated detection and removal of artifacts. There are a variety of algorithms, based on either statistical thresholding, either fixed or adaptive, or using Bayesian approaches. Individual level ICA based on Blind Source Separation (BSS) seems to have established a dominant position for removal of various types of fixed‐source artifacts (Delorme et al., 2007; Nolan et al., 2010), with several methods for automated artifact IC detection (Nolan et al., 2010; Pion‐Tonachini et al., 2019). Recent complementary methods such as Artifact Subspace Reconstruction (ASR) propose solutions to remove transient large amplitude noise from the data (Chang et al., 2019). Although many automated artifact removal techniques still require visual confirmation, fully automated algorithms may actually be in good agreement with visual inspection for high density recordings (Hatz et al., 2015). This opens up possibilities for large‐scale endeavors such as ENIGMA‐EEG to implement fully automated pipelines such as the one implemented by one of us (SJB) (https://github.com/sjburwell/eeg_commander) and others (https://www.frontiersin.org/articles/10.3389/fnins.2018.00097/full; https://www.frontiersin.org/articles/10.3389/fninf.2015.00016/full). We note, however, that there is no agreed upon gold standard for automated artifact removal yet.

Finally, our large datasets allow us to quantitatively investigate the effect of data quality on some of the phenotypes that were collected in the population‐based and clinically ascertained samples. Meta‐data about the cleaning process—for example, data recording length, number of channels lost, or the number of epochs rejected after visual cleaning—could all be used to predict, for example, the age of the subject, or any psychiatric or behavioral outcome. As such variables of recording and processing quality may be associated with phenotypes, this information could be invaluable to the whole field of EEG and possibly result in specific thresholds for acceptable data.

### 4.4 Cohort sampling consistency

Age, ancestry, ascertainment, and disease status all play major roles in heterogeneity across our cohorts. Table 1 shows an overview of the cohorts currently contributing to ENIGMA‐EEG. Sampling variability arguably leads to problems when meta‐analyzing results and could lead to reduced power. EEG features change substantially with age (Niedermeyer, 1999b). The power of oscillations at specific frequencies may reduce by as much as 10 dB (i.e., a 67% decrease in amplitude) *on average* from childhood to adulthood (Vandenbosch et al., 2019). Theta band oscillations show the most extreme change, but alpha, beta, and gamma changes are observed as well as changes in alpha peak frequency (Benninger et al., 1984; Gasser et al., 1988; Marshall et al., 2002; Vandenbosch et al., 2019). Concurrent changes are seen for other derived EEG features, such as sensor‐level connectivity and graph parameters (Boersma et al., 2011; Smit & Anokhin, 2016; Smit et al., 2010, 2012, 2016).

Developmental and age‐related changes do not necessarily mean that different genes determine individual differences at different ages. However, participants differ in the speed in which their functional brain activity matures, and this difference is heritable (Vandenbosch et al., 2019). It is also evident that developmental changes occur with variable rate across time and space (Niedermeyer, 1999b). For example, communication between brain areas changes such that qualitatively different patterns in the functional connectivity network appear, changing from a relatively random to a more ordered network structure (Boersma et al., 2011; Smit & Anokhin, 2016). During the same developmental period, the network topology changes in the minimum spanning tree parameters of graph diameter and maximum centrality (Tewarie et al., 2015).

In addition, it has become clear that gene expression changes drastically during development, possibly to promote appropriate maturation of the brain and other tissues. One of the gene‐regulating processes, methylation, shows well‐timed changes that allow the prediction of a subject's age (Bocklandt et al., 2011; Dongen et al., 2016; Hannum et al., 2013; Simpkin et al., 2017); recent work by ENIGMA's Epigenetics group has linked ongoing methylation to hippocampal volume and other features of brain morphometry (Jia et al., 2019). Such changes imply that different genes play a role across developmental age groups. These observations indicate that age is likely to induce heterogeneity across cohorts with age differences and that particular care must be taken when including childhood samples. Additionally, sex differences in developmental genetic association studies of both resting‐state EEG coherence and event‐related oscillations have been reported (Chorlian et al., 2017; Meyers et al., 2019), consonant with other developmental genetic studies (Cousminer et al., 2014). EEG features may also be modulated by different stages of neurological and psychiatric diseases, and these may impact the comparability of EEG recordings obtained from patients with the same disorder at different stages of disease progression (Douw et al., 2019).

Some cohorts in ENIGMA‐EEG have multiple recordings of their subjects in partial longitudinal study designs. Combined longitudinal/cross‐sectional designs allow investigation of age modulation of genetic risk. For example, the detrimental effect of the apolipoprotein E epsilon 4 (APOE4) allele and the protective effect of the epsilon 2 (APOE2) allele on the brain can be investigated using such age modulation models. Multiple observations per subject increases power and reduces confounding. However, the numbers do not quite reach those required to perform genome‐wide longitudinal genetic testing. Consistent with other genome‐wide studies, we therefore opted to start out by selecting a single observation per individual for the first runs of analyses. We hope and expect that with the increased availability of EEG data in people with genetic profiles, this will change in the future.

Large genetic studies have mainly focused on cohorts of European descent. This European Ancestry bias is not unique to many of the cohorts in ENIGMA‐EEG, but systemic within the GWAS literature (Peterson et al., 2019). Although the proportion of studies including individuals of diverse ancestry has been increasing with several ENIGMA‐EEG cohorts including non‐European individuals (Meyers et al., 2017), this remains a critical issue that the field must address (Popejoy et al., 2020).

In summary, to allow reliable meta‐analysis of EEG genetic association study data in ENIGMA, we encourage researchers to use 64 lead recordings or more, use automated cleaning and QC procedures, and perform sensitivity analyses to recording/analysis choices in EEG parameter extraction to safeguard homogeneity across cohorts. Additional phenotyping in the form of (family) history of neurological disorders, psychiatric disorders, and substance use as well as measures of social‐economic status and educational attainment (Abdellaoui et al., 2019) would greatly help in providing additional covariates for the association analyses. EEG should be measured in sufficient duration to yield reliable estimates. But since many oscillatory parameters, even when measured over relatively short periods, are strongly heritable (Linkenkaer‐Hansen et al., 2007; Smit et al., 2005, Smit et al., 2012), this indicates that they generally are reliably estimated. We note, however, that for measures dependent on dynamics changes in oscillatory activity, such as vigilance, longer periods are needed to establish reliable estimates (Jawinski et al., 2018).

## 5 NEXT STEPS FOR ENIGMA‐EEG

There is renewed interest in collecting EEG in large cohort studies. Several cohort studies within ENIGMA‐EEG have initiated the collection of EEG recordings in samples of over 1,000 participants, using newer EEG equipment and higher density electrode montages. These include cohorts from Germany (LIFE cohort) and the United States (The Tennessee Synchrony & Speech Cohort). These high‐quality, high‐density recordings provide additional opportunities to investigate the relation between EEG and psychiatric/neurological disorders. Most of the ENIGMA‐EEG cohorts are population‐based samples, while some are samples ascertained for psychiatric disorders or epilepsy.

### 5.1 Future measures

EEG research is increasingly mapping oscillatory function to biological and neurological mechanisms, with complex interactions across space and frequency that subserve the integration of information in a hierarchically organized brain (Bonnefond et al., 2017; Canolty & Knight, 2010; Jensen & Colgin, 2007; Tingley et al., 2018). Oscillations at different frequencies are increasingly understood not to have a one‐to‐one mapping with function (Wolfgang Klimesch, 1999). Multiple functions may be present in oscillations, such as the multiple function linked to alpha oscillations: inhibition of sensory information during visual processing (Jensen et al., 2012; Jensen & Mazaheri, 2010; Klimesch et al., 2007; Yao et al., 2019), default mode function (Laufs et al., 2003; Mantini et al., 2007), and cortico‐subcortical communication (Horschig et al., 2015).

Oscillatory activity may be more directly linked to synaptic function than more indirect measures from imaging modalities based on energy expenditure, such as functional MRI or ^18^F‐fluorodeoxyglucose (FDG) PET. As indicated above, prior studies have linked beta oscillations to GABA alpha receptor subunit genes in alcohol use disorders (Edenberg et al., 2004; Porjesz et al., 2002). Other studies have highlighted the role of GABA interneurons for various EEG oscillations in schizophrenia (Edden et al., 2009; Rowland et al., 2013). Investigating the ties between oscillations and synaptic function may complete the circle, linking genetic variants to behavioral disorders to brain function.

Among our next endeavors are genome‐wide scans of measures of oscillation‐based communication between distant brain areas (Fries, 2005; Stam, 2014) and oscillation dynamics (Linkenkaer‐Hansen et al., 2001). The importance of neural communication for behavior and behavioral disorders is well documented (Paus et al., 2008; Uhlhaas & Singer, 2010). EEG is widely used for establishing functional connectivity and yields a wealth of information on the synchrony between distant brain areas (Lobier et al., 2014; Nolte et al., 2004; Stam & van Dijk, 2002; Stam et al., 2007). The brain is a highly organized, nonrandom network that balances substantial wiring costs with enhanced communication capacities (Bullmore & Sporns, 2009, 2012; Stam, 2014). This optimization is obtained by a modular community structure with an uneven importance distribution across the nodes (van den Heuvel & Sporns, 2013). Areas of high importance (“hubs,” or highly central nodes) are particularly vulnerable to impairments causing large dysfunctions (Heuvel et al., 2013; Stam et al., 2009). The goal of ENIGMA‐EEG is to elucidate how genetic variants influence communication between brain areas and the connectivity patterns of the network, matching those variants to neurological and psychiatric disorders.

Our ongoing investigation of functional connectivity is based on detecting statistical patterns across a selection of EEG signals using coherence. Since coherence is well known to show spurious connectivity due to volume conduction effect (i.e., high coherence is expected for subjects with strong, deep oscillatory sources), we used local bipolar derivations as a means to reduce this effect. Similar to current source density (Babiloni et al., 2001; Hjorth, 1975; Nunez & Westdorp, 1994), local bipolar derivations are proportional only to local currents (Yao et al., 2019). Our procedure closely follows a recent GWAS by one of our groups (Meyers et al., 2020), approximately doubling the sample size.

Temporal dynamics of oscillatory activity are a window into a brain that keeps itself in an equilibrated state where activity neither dies out quickly over time nor avalanches into uncontrolled spiking activity. Such states are generally obtained via self‐organization, balancing excitatory and inhibitory neuronal activity (Atallah & Scanziani, 2009; Bak et al., 1987; Ferguson & Gao, 2018; Levina et al., 2007; Selten et al., 2018). It has been shown that this balancing leads to maximal representational capacity of the neural network (Kinouchi & Copelli, 2006). Temporal correlations in the oscillatory activity of the brain reflect this balanced state (Linkenkaer‐Hansen et al., 2001; Poil et al., 2012), but also show quite some variation in the particular tuning that result in variable levels in the signal autocorrelation. These variable levels reflect the brain's tendency for faster or slower state switching, with consequences for behavior (Palva et al., 2013; Prent & Smit, 2019; Smit et al., 2013) and psychopathology (Linkenkaer‐Hansen, 2005; Montez et al., 2009; Moran et al., 2019; Nikulin et al., 2012). These fast or slow decaying temporal correlations are measurable in EEG, show large individual variation, and are heritable (Linkenkaer‐Hansen et al., 2007). Our goal will be to elucidate how genetic variants affect this oscillatory balance and determine whether these variants are part of excitatory and inhibitory synaptic functioning (such as glutamate and GABA receptor genes). We will investigate whether temporal dynamics vary for participants with a high genetic liability for neurological disorders such as epilepsy, but also for participants with high sensory sensitivity complaints in, for example, autism spectrum disorder (American Psychiatric Association, 2013; Robertson & Baron‐Cohen, 2017) and tinnitus (Hébert et al., 2013).

Our very large EEG database allows us to not just investigate the genetics of EEG parameters, but also to plot normative developmental curves across the wide age range available in our datasets (Table 1), possibly extended with other developmental samples (Anokhin et al., 2017; Obeid & Picone, 2016; Smit & Anokhin, 2016). These data are valuable to investigate neurodevelopmental disorders and deviant brain development, such as ADHD and autism, with ample power to detect differences. Further, polygenic risk scores based on the ENIGMA‐EEG discovery genome‐wide association meta‐analysis (GWAMA) can be constructed to provide liability indices that may be associated with mental disorders, individual differences in cognition, brain development, and connectivity patterns. The advantage of such an approach is that the subjects reflect the full range of individual variation across the population (Martin et al., 2018; Simmons & Quinn, 2014).

### 5.2 Future methods

Multiple aspects of these new scientific ventures with EEG recordings may prove useful for clinical purposes. With the advent of big data and the successful application of machine learning techniques, EEG research can start measuring up with other imaging modalities to perform disease classification and treatment outcomes. These predictive techniques are maturing quickly (Janssen et al., 2018). In fMRI research, imaging the activity of the brain pretreatment can successfully predict electroconvulsive treatment (ECT) outcome for otherwise treatment‐refractory depressed patients (Waarde et al., 2015). Bridging such findings to EEG research will require novel designs in artificial neural networks tuned to the specific spatio‐temporal aspects of EEG oscillations (Schirrmeister et al., 2017). These have so far largely been developed for detecting epileptic seizures, sleep staging, and brain computer interfacing (BCI (Ding et al., 2015)). We foresee an expansion of such models to many other areas of behavioral (dys)function.

Pharmaco‐EEG can be used to evaluate drug targets and for drug repurposing. For example, mecamylamine has recently been used as an Alzheimer's disease model (Simpraga et al., 2018). The described changes in behavior—as well as changes in EEG oscillations induced by mecamylamine—are highly reminiscent of AD, but are fully reversible. Although the effectiveness of drugs reversing the effects of such models is debatable, the systematic use of EEG during Phase II clinical trials could help in establishing a database that marks neuronal changes induced by drugs. This, in turn, could help in repurposing drugs for neurological and psychiatric disorders by investigating how changes in EEG patterns are resolved (Jobert et al., 2012).

### 5.3 Future‐omics

Genomics of human complex trait variation may be a first step in understanding the genetics underlying human trait variation. In the future, we wish to explore other types of variation, for example, due to rare variants or to other types of structural variants, affecting brain function. Genetic studies addressing these traits are increasingly considering other ‐omics levels to address variation and the pathways between genotype and phenotype. Methylation studies for cognition and educational attainment (Dongen et al., 2018; Linnér et al., 2017) have uncovered multiple genome‐wide significant differences in methylation at CpG sites. Genome‐wide testing of epigenetic marks has been explored within ENIGMA for subcortical volumes; differentially methylated regions in the genome were suggested to be associated with hippocampal volume (Jia et al., 2019). DNA methylation at these loci affected expression of proximal genes among other traits in learning and memory (Jia et al., 2019). Other ‐omics that are promising include transcriptomics and metabolomics (van der Lee et al., 2018), possibly combined into multi‐omics approaches (Wu et al., 2018). We feel that these techniques are especially suitable for the investigation of changes in brain maturation, behavioral, development, and decline or resilience to decline in older age.

## 6 CONCLUSION

In ENIGMA‐EEG, we expect that large‐scale studies of EEG data will help to elucidate the causal mechanisms of liability genes affecting the functioning brain, by identifying the genetics of EEG features. Given the wealth of EEG data available worldwide, and the promise of other imaging modalities such as structural measures of fMRI in massive data collections such as available in the UK Biobank (Manolio et al., 2012), there is still a huge incentive to collaborate across cohorts that have collected EEG and genetic data to combine their efforts and reach ever increasing sample sizes that have proven so useful for other fields (Sullivan, 2010; Sullivan et al., 2017). Such multisite and international alliances can boost power and may also help in avoiding the small sample pitfalls that sometimes may have stalled progress in areas of human neuroscience (Button et al., 2013).

What ENIGMA‐EEG will be doing in the near future is to expand the investigations to increasingly complex EEG biomarkers and diving ever more deeply into the functioning brain. Ever increasing sample sizes will help us in finding more genetic variants affecting brain activity—most likely a growing set that includes both common and rare variants, as well as structural variation. The growing sample sizes, analyzed using harmonized protocols, should also increase our power to find significant genetic correlations with behavioral traits, and further our understanding of the effect of neurological, psychiatric, and other liability genes on brain function. We therefore call on additional cohorts with EEG and whole‐genome scans to join our effort. This can be done by simply emailing the first author (D.S.) or via the ENIGMA‐EEG website (http://enigma.ini.usc.edu/ongoing/enigma‐eeg‐working‐group/).

## CONFLICT OF INTEREST

T.E. has received speaker's fee from Lundbeck AS. O.A.A. reports personal fees from Lundbeck and from HealthLytix. P.M.T. received a research grant from Biogen, Inc., for work unrelated to this manuscript.

## ETHICAL APPROVAL

Not applicable.

### PEER REVIEW

The peer review history for this article is available at https://publons.com/publon/10.1002/brb3.2188.

## Supporting information

Supplementary Material

## Data Availability

ENIGMA‐EEG results are available for download at http://enigma.ini.usc.edu/ongoing/enigma‐eeg‐working‐group/. The protocols can be found on Github at dirkjasmit/ENIGMA‐EEG.
