# Supplementary material for: Large‐scale collaboration in ENIGMA‐EEG: A perspective on the meta‐analytic approach to link neurological and psychiatric liability genes to electrophysiological brain activity
Source: Brain Behav. 2021 Jul 21;11(8):e02188. doi: 10.1002/brb3.2188 (PMC8413828; doi:10.1002/brb3.2188)
Supplement: Supplementary file 1 — Supplementary Material [file BRB3-11-e02188-s001.docx]

**SUPPLEMENTARY INFORMATION**

**ENIGMA Coherence Analysis Frequency Band Selection (David Chorlian)**

*Choice of Frequency Bands for bipolar EEG coherence analysis*

Rather than rely only on the conventional band structure of EEG and knowing of no discussion of this issue

based on large data sets, we used EEG coherence results available at the Henri Begleiter Neurodynamics

Laboratory at SUNY Downstate Health Sciences University to investigate this topic. We examined the topic

from two points of view: the descriptive, based on a study of a large number of observations with an age

range of 7 to 70 using the conventional band structure of EEG to examine frequency differences based on

age, sex and topography; and the analytic, based on the study of a relatively small number of observations

for which we had data in .5 Hz bins extending from 3 to 28 Hz with a view to modeling the ability of bands

to reproduce the important frequency specific features of the unbanded data.

*Descriptive Perspective*

Age, sex, and topographical variation in bipolar coherence was obtained from the large COGA sample (N >

12000 observations with an age range of 7 to 70), portions of which have been discussed in previous papers

on heritability [4] and on a GWAS [1], as well as in relation to polygenic risk scores (PRS) of psychiatric

illness [2]. Bipolar derivations were used to calculate coherence to obviate volume conduction effects because

many of our earlier recordings were obtained using only 21 electrodes from the 10-20 system, and we wished

to obtain continuity with our earlier recordings in subsequent analyses. The available data had 27 coherence

pairs. The essence of our results are shown in figure 1, which illustrates the age specific means of coherences

in low theta (3-5 Hz), low alpha (7-9 Hz), and high alpha (9-12 Hz) for both interhemispheric (top panel)

and intrahemispheric coherence (bottom panel). For all 4 coherence pairs, trajectories of low and high alpha

coherence exhibited varying relations over age, strongly suggesting the value of the separation of the two

bands in our planned analysis. (It should be noted that the age distribution of the data is far from uniform

with far more observations per age range for the younger ages (<= 30) than for the older ages.)

*Analytic Perspective*

The ability of bands to reproduce the important frequency specific features of the unbanded data was

assessed by finding a vector of frequency separation points which applied to the unbanded data produced

a band structured data set which was such that a linear combination of the banded data was best able to

reconstruct the unbanded data, as measured by a relative matrix distance (Frobenious distance) between

the unbanded data and the reconstructed data.

For this analysis, a data set comprising 240 SUNY subjects (120 clean controls; 120 AUD; age matched

between 30 and 50 years of age) with individual frequency bins (.5 Hz) recorded from a large number of

coherence pairs. (Some of this data was used in [3].) The pairs selected for analysis are the same 27 pairs

as in the COGA data described above; a somewhat different set of 23 pairs based on a plausible minimal

channel list also chosen from the 10-20 system was also analyzed. The results of that analysis provided an

almost identical separation vector to that found using the 27 pairs.

A discrete algorithm based on the Nelder-Mead function minimization procedure was used to identify separation

points for optimal selection of frequency bands by the criterion of providing the best reconstruction

points averaged over all of the subjects included in the data set. The algorithm requires an initial choice of

separation points, which are then changed according to the criterion described in the previous sentence. The

algorithm is simplistic and it is possible the solution will been trapped in a local optimum determined by the

initial choice of separation points. However, examination of the results based on different initial choice of

separation points gave consistent final separation points. The frequency range was 3 Hz to 27 Hz (49 bins).

The objective function to find a measure of the reconstruction error uses Matlab linsolve() to solve A * X =

B where B is the matrix consisting of all the individual coherence values (number of coherence pairs rows

by number of frequency bins) stacked on top of each other and A is the matrix consisting of all the means

of the individual coherence values defined by the ranges of frequencies to be combined (number of coherence

pairs rows by number of frequency bands) stacked on top of each other. Then the Frobenious norm of the

matrix B - A * X, which represents the difference between the observed individual frequency values and

those given by representation of them in terms of the values of the means of the frequency bands, divided

by the Frobenious norm of the matrix B, represents a measure of (proportional) error.

The result of this calculation for using either 5 or 6 bands (4 or 5 separation points) were the following

separation points:

7.5 10.5 12.5 17.5 22.5

8.0 10.5 13.0 20.5

Note that both divisions of the spectrum differentiate low and high alpha, as is clearly desired given the

trajectory plots.

*Bibliography*

[1] A genome-wide association study of interhemispheric theta EEG coherence: implications for neural

connectivity and alcohol use behavior. Meyers JL, Zhang J, Chorlian DB, Pandey AK, Kamarajan C, Wang

JC, Wetherill L, Lai D, Chao M, Chan G, Kinreich S, Kapoor M, Bertelsen S, McClintick J, Bauer L,

Hesselbrock V, Kuperman S, Kramer J, Salvatore JE, Dick DM, Agrawal A, Foroud T, Edenberg HJ, Goate

A, Porjesz B. Mol Psychiatry. 2020 May 20. doi: 10.1038/s41380-020-0777-6.

[2] Association of Polygenic Liability for Alcohol Dependence and EEG Connectivity in Adolescence and

Young Adulthood. Meyers JL, Chorlian DB, Johnson EC, Pandey AK, Kamarajan C, Salvatore JE, Aliev F,

Subbie-Saenz de Viteri S, Zhang J, Chao M, Kapoor M, Hesselbrock V, Kramer J, Kuperman S, Nurnberger

J, Tischfield J, Goate A, Foroud T, Dick DM, Edenberg HJ, Agrawal A, Porjesz B. Brain Sci. 2019 Oct

17;9(10):280. doi: 10.3390/brainsci9100280. PMID: 31627376

[3] EEG coherence: topography and frequency structure. Chorlian DB, Rangaswamy M, Porjesz B. Exp

Brain Res. 2009 Sep;198(1):59-83. doi: 10.1007/s00221-009-1936-9. Epub 2009 Jul 22. PMID: 19626316

[4] Heritability of EEG coherence in a large sib-pair population. Chorlian DB, Tang Y, Rangaswamy

M, O’Connor S, Rohrbaugh J, Taylor R, Porjesz B. Biol Psychol. 2007 Jul;75(3):260-6.

doi: 10.1016/j.biopsycho.2007.03.006. Epub 2007 Apr 5. PMID: 17498861
